# Proteomic Analysis Reveals a Critical Role of the Glycosyl Hydrolase 17 Protein in *Panax ginseng* Leaves under Salt Stress

**DOI:** 10.3390/ijms24043693

**Published:** 2023-02-12

**Authors:** Ju-Young Jung, Cheol Woo Min, Jeong Woo Jang, Ravi Gupta, Ji-Hyun Kim, Young-Hun Kim, Sung Won Cho, Young Hun Song, Ick-Hyun Jo, Randeep Rakwal, Yu-Jin Kim, Sun Tae Kim

**Affiliations:** 1 Department of Plant Bioscience, Pusan National University, Miryang 50463, Republic of Korea; 2 College of General Education, Kookmin University, Seoul 02707, Republic of Korea; 3 Department of Life Science and Environmental Biochemistry, Pusan National University, Miryang 50463, Republic of Korea; 4 Department of Agricultural Biotechnology, Seoul National University, Seoul 08826, Republic of Korea; 5 Department of Herbal Crop Research, Rural Development Administration, Eumseong 369-873, Republic of Korea; 6 Faculty of Health and Sport Sciences, University of Tsukuba, 1-1-1 Tennodai, Tsukuba 305-8574, Japan; 7 Research Laboratory for Biotechnology and Biochemistry (RLABB), GPO 13265, Kathmandu 44600, Nepal

**Keywords:** *Panax ginseng*, salt stress, shotgun proteomics, glycosyl hydrolase 17, overexpression

## Abstract

Ginseng, an important crop in East Asia, exhibits multiple medicinal and nutritional benefits because of the presence of ginsenosides. On the other hand, the ginseng yield is severely affected by abiotic stressors, particularly salinity, which reduces yield and quality. Therefore, efforts are needed to improve the ginseng yield during salinity stress, but salinity stress-induced changes in ginseng are poorly understood, particularly at the proteome-wide level. In this study, we report the comparative proteome profiles of ginseng leaves at four different time points (mock, 24, 72, and 96 h) using a label-free quantitative proteome approach. Of the 2484 proteins identified, 468 were salt-responsive. In particular, glycosyl hydrolase 17 (*PgGH17*), catalase-peroxidase 2, voltage-gated potassium channel subunit beta-2, fructose-1,6-bisphosphatase class 1, and chlorophyll a-b binding protein accumulated in ginseng leaves in response to salt stress. The heterologous expression of *PgGH17* in *Arabidopsis thaliana* improved the salt tolerance of transgenic lines without compromising plant growth. Overall, this study uncovers the salt-induced changes in ginseng leaves at the proteome level and highlights the critical role of *PgGH17* in salt stress tolerance in ginseng.

## 1. Introduction

Soil salinity is a major challenge to modern agriculture that adversely affects crop growth, productivity, and quality [1]. A high salt concentration causes water uptake deficiency and nutrient imbalances in plants, and induces osmotic and ionic stresses [2,3]. Moreover, high salinity also leads to physiological changes, growth inhibition, and developmental changes by inhibiting photosynthetic activity and modulating cell expansion through complex cellular signaling [3,4]. Therefore, understanding the salt-responsive mechanisms involved is an important issue for crop production. 

Among the different crops cultivated globally, traditional medicinal plants are also the focus of farmers because they are utilized widely as remedies for human diseases and for their perceived health benefits [5]. Ginseng (*Panax ginseng* Meyer) has been used for thousands of years in East Asian countries due to its many active constituents, including the well-reported ginsenosides [6]. More than 200 ginsenosides have been identified and characterized for their appreciable pharmacological and medicinal properties, such as anti-cancer, anti-inflammatory, anti-obesity, anti-diabetes, and anti-cardiovascular activities [7]. Although the therapeutic potential of ginseng has been widely reported, numerous challenges remain in improving its cultivation methods and productivity, particularly during the occurrence of abiotic stresses [7]. 

Ginseng is a perennial plant that requires at least four to six years of cultivation to allow the sufficient accumulation of ginsenosides [8]. On the other hand, rapid climatic changes and effects, coupled with excessive use of chemical fertilizers, have resulted in soil salinization in ginseng fields [9]. Ginseng, a salt-sensitive crop, can tolerate soil salinity up to 0.5 ds/m, after which a drastic change in the ginseng yield is noted [10]. A high salt salinity reduces plant water uptake, resulting in nutrient and ion imbalances [2,3]. Moreover, high salinity also leads to the accumulation of reactive oxygen species (ROS), which causes the inhibition of enzyme activities, reduced carbon-use efficacy, and the decomposition of membrane structures [11,12,13]. Furthermore, salt stress inhibits photosynthesis significantly, which is a major cause of decreased ginseng production [3,4,14]. Therefore, efforts are needed to understand the salt-induced changes in crops and to identify potential candidates that can be targeted in the future to develop salt-stress-tolerant crops. 

Recent advances in proteomic analysis tools have facilitated the identification of critical proteins that respond to stress conditions [15]. Previous studies on the identification of salt-responsive proteins in ginseng used a gel-based proteomics approach, which resulted in the identification of only a few potential candidate proteins [16,17]. Moreover, these studies only identified high-abundance proteins, limiting the understanding of global changes in response to salt stress in ginseng. This study used a high-throughput proteomic approach to evaluate the effects of salt stress on ginseng leaves in a time-dependent manner. Among the different salt-induced proteins identified in this study, functional characterization and validation of glycosyl hydrolase 17 (PgGH17) proteins was performed. The heterologous expression of the *PgGH17* in *Arabidopsis thaliana* improved the salt stress tolerance of transgenic lines, confirming a crucial role of PgGH17 in the salt stress response.

## 2. Results

### 2.1. Physiological and Enzymatic Changes in Ginseng Leaves during Salt Stress

**Figure 1:**
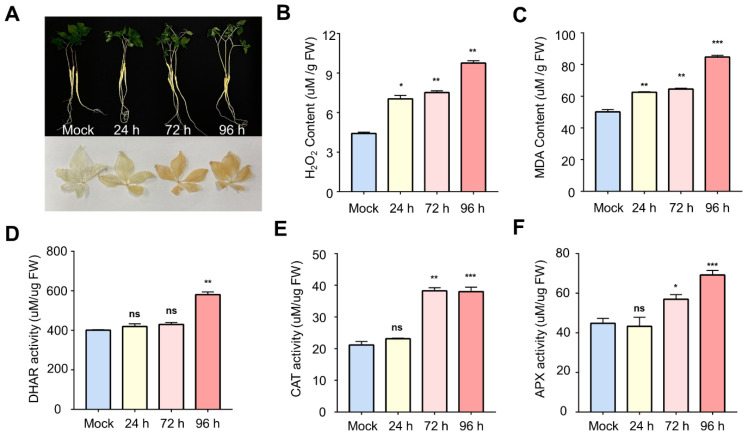
(**A**) Effect of salt stress on ginseng (upper panel) and accumulation of ROS as observed by DAB staining (lower panel). Measurement of (**B**) H_2_O_2_, and (**C**) lipid peroxidation contents and antioxidant enzyme activities of (**D**) DHAR, (**E**) CAT, (**F**) APX at Mock, 24 h, 72 h, and 96 h following salt stress. The bars show mean values ± standard deviations summarized from individual values in independent experiments (*n* = 3). Significant differences between mock and salt-treated samples are highlighted by an asterisk. (ns indicates not significance, * indicates significance of *p* < 0.05, ** indicates significance of *p* < 0.01, *** indicates significance of *p* < 0.001).

The phenotypic changes in ginseng during salt stress were examined by exposing two year-old ginseng plants to 250 mM NaCl treatment, and the accumulation of ROS was monitored by 3,3′-diaminobenzidine (DAB) staining. Ginseng plants exhibited a severe wilting phenotype and the gradual accumulation of H_2_O_2_ in their leaves in response to salt stress (Figure 1A). The H_2_O_2_ content was calculated to be 4.44, 7.12, 7.46, and 9.69 μmol/g in the control, at 24, 72, and 96 h, respectively (*p*-value < 0.01), indicating 120% H_2_O_2_ accumulation in the 96 h sample compared to the control (Figure 1B). In parallel to the H_2_O_2_ accumulation, a high level of lipid peroxidation was also observed: 50.13, 62.52, 64.52, and 84.71 μmol/g in the control, at 24, 72, and 96 h, respectively (*p*-value < 0.001) (Figure 1C). Activity analysis of the antioxidant enzymes, including dehydroascorbate reductase (DHAR), catalase (CAT), and ascorbate peroxidase (APX), showed 45% (400.71 to 580.71 μmol/g), 80% (21.15 to 37.97 μmol/g), and 31% (44.78 to 58.95 μmol/g) higher DHAR, CAT, and APX activities in 96 h salt-treated sample than in the control (Figure 1D–F). These results confirm the over-accumulation of ROS and subsequent lipid peroxidation in the salt stress-treated ginseng leaves, and the subsequently increased activities of antioxidant enzymes, which is a hallmark effect of salt stress on plants. The downstream high-throughput proteomic analysis was carried out after validating the effect of salt stress by physiological and enzymatic analyses.

### 2.2. Label-Free Proteomic Analysis of Salt Treated Ginseng Leaves

**Figure 2:**
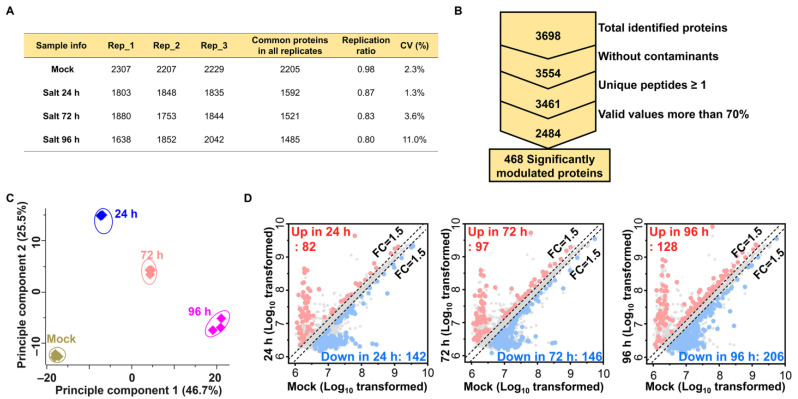
Proteomic analysis of ginseng leaves in response to salt stress treatment for 24 h, 72 h, and 96 h. (**A**) Overview of the proteins identified in three biological replicates of ginseng samples treated with different durations of salt stress. (**B**) The distribution of the total identified and significantly modulated proteins. (**C**) Principal component analysis showing a clear separation of significantly modulated proteins. (**D**) Volcano plots highlighting more than 1.5-fold relative fold changes among the four samples.

**Figure 3:**
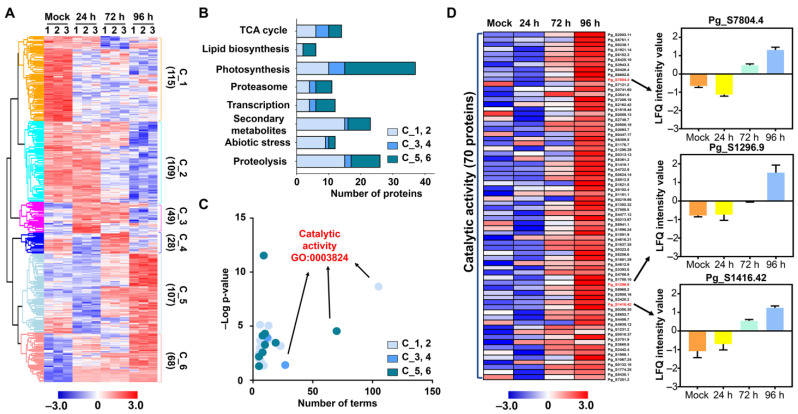
Multivariate analysis and functional classification of differentially modulated proteins (DMPs) in salt-treated ginseng leaves. (**A**) HCA grouped all 468 DMPs into 6 clusters based on their abundance patterns. (**B**) Functional overview of proteins classified into clusters using MapMan software. (**C**) GO enrichment analysis of proteins corresponding to their molecular functions in all clusters. (**D**) Heatmap showing the fold-change variation of each protein, corresponding to catalytic activity, and a box chart highlighting the 3 proteins predicted to have potential salt tolerance, with increased abundance in salt-treated ginseng leaves.

The salt stress-induced proteome alterations and salt-responsive proteins were examined by a proteome analysis of ginseng leaves using a label-free quantitative proteomic approach, liquid chromatography–mass spectrometry (LC–MS), which led to the identification of 28,313 peptides and 12,288 unique peptides that matched 3698 protein groups (Figure 2A,B). Of the 3698 proteins, 2484 were marked as potential contaminants and were removed, along with the proteins showing missing values of less than 70% across the three replicates in each sample (Figure 2B). Variation across the sample sets was examined, and a principal component analysis (PCA) was performed that showed a clear separation of the 72 h and 96 h salt-treated sample sets from the control sample in principal component 1, which accounted for 46.7% of the total variation, while a 24 h sample set was separated from the control in principal component 2 that accounted 25.5% of the total variation (Figure 3C). The variable importance in projection (VIP) score plot revealed the top 15 proteins that contributed to the total variation in the partial least squares discriminant (PLSD) plot, including D-mannose-binding lectin, proteasome subunit beta 1, photosystem II PsbP, and tubulin (Figure S1). ANOVA identified 468 salt-stress-responsive proteins in ginseng leaves (Figure 2B,D and Figure 3A). Hierarchical clustering analysis (HCA) classified these salt stress-responsive proteins into six clusters, numbered Clusters 1–6, containing 115, 109, 49, 28, 107, and 68 proteins, respectively. Of these, the proteins involved in C_3, 4, 5, and 6 showed an increased abundance in the 24, 72, and 96 h samples compared to the control, while proteins of C_1 and 2 showed mixed abundance profiles in the mock and 24 h samples (Figure 3A).

### 2.3. Functional Classification of Identified DMPs

MapMan analysis was carried out to gain further insight into the functions of the identified salt stress-responsive proteins or differently modulated proteins (DMPs). The results showed that the identified DMPs were mostly associated with proteolysis, abiotic stresses, secondary metabolites, transcription, the proteasome, photosynthesis, lipid biosynthesis, and the tricarboxylic acid (TCA) cycle (Figure 3B). In particular, 15, 16, and 8 DMPs from C_1 and C_2 were associated with proteolysis, secondary metabolites, and abiotic stresses, respectively, including peptidase M3, serine-type endopeptidase, adenosine triphosphate-dependent peptidase, uridine diphosphate-glucosyl transferase (UGT), and beta-amylin synthase (Figure 3B). In addition, C_3 and C_4 DMPs were dominated mainly by proteins related to the TCA cycle and photosynthesis, such as fumarate hydratase and ATP citrate synthase, plastocyanin 1 (PETE1), ferredoxin A (FED A), and light-harvesting complex associated with photosystem (LHCA) (Figure 3B). In addition, 175 proteins (involved in C_5 and C_6) with increased abundance in the 96 h sample were associated with photosynthesis, the proteasome, proteolysis, and lipid biosynthesis (Figure 3B).

Further gene ontology (GO) enrichment analysis using AgriGO v2.0 showed that 105, 27, and 70 DMPs were related to the catalytic activity (GO:0003824) in C_1/2, C_3/4, and C_5/6, respectively, in the molecular function category (Table S2). In the case of C_1/2, 105 DMPs (47%) were associated with various catalytic activities, including oxidoreductase, lyase, structural molecular, ligase, transferase, ATPase, peptidase, and isomerase activities. Only 27 DMPs (35%) associated with catalytic activity were identified (Table S2). In parallel to C_1/2, 70 DMPs (49%) involved in C_5/6 had catalytic activities, including oxidoreductase, peptidase, isomerase, and ATPase (Table S2).

### 2.4. Validation of Candidate Proteins Using qRT-PCR

**Figure 4:**
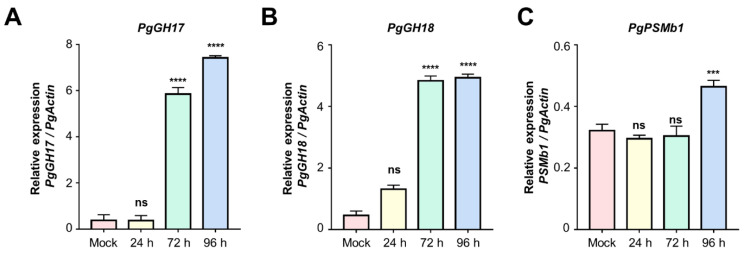
Bar charts showing the mRNA expression levels of *PgGH17, 18*, and *PgPSMb1* genes in mock, 24 h, 72 h, and 96 h salt-stress-treated ginseng leaves, as measured by qRT-PCR analysis. The data are represented by the means of at least three replicates, and significant differences are indicated between the mock and salt-treated samples. (ns indicates no significance, *** indicates significance of *p* < 0.001, **** indicates significance of *p* < 0.0001).

Among the highly salt-stress-induced proteins, nine proteins, including glycosyl hydrolase (GH) family proteins (17, 18), proteasome subunit beta 1 (PgPSMb1), pyruvate kinase, D-mannose lectin C-terminal domain, leucine-rich repeat (LRR) family protein, fasciclin-like arabinogalactan protein 10, prostatic steroid-binding protein (PsbP) domain-containing protein 3 and aspartyl protease family protein, were selected for validation at the RNA level by using quantitative real-time (qRT)-PCR analysis. Among these proteins, the expression levels of three genes, including *PgGH17* and *PgGH18*, showed significantly higher induction in ginseng leaves during salt stress conditions at later time points (72 and 96 h), while *PgPSMb1* was induced in 96 h samples compared to controls (Figure 4A–C). In particular, *PgGH17* showed 19.52-fold increased expression in salt stress-treated ginseng leaves compared to the control (Figure 4A). A previous study also reported the salt-induced accumulation of GH17 [17]. Therefore, GH17 was selected, in order to investigate its potential role in salt stress tolerance.

### 2.5. Sequence Analysis and Cloning of PgGH17

**Figure 5:**
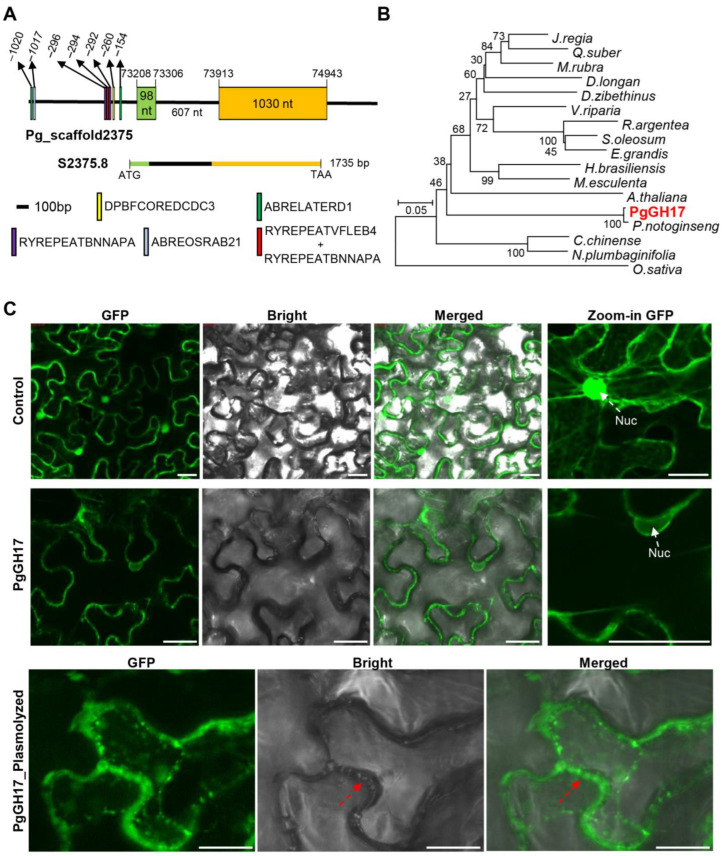
Cloning and overexpression of *PgGH17* in *Arabidopsis*. (**A**) Gene structure of PgGH17 in the ginseng genome. (**B**) Phylogenetic analysis of PgGH17. The PgGH17 protein and homologous proteins from various plants, including *Juglans regia* (XP018825764.1), *Quercus suber* (XP023873688.1), *Morella rubra* (KAB1213853.1), *Durio zibethinus* (XP022760689), *Dimocarpus longan* (AKE49478.1), *Hevea brasiliensis* (XP0216687), *Manihot esculenta* (XP02160835), *Vitis riparia* (XP034684864.1), *Rhodamnia argentea* (XP0305279), *Syzygium oleosum* (XP030455949), *Eucalyptus grandis* (XP0100635), *Panax notoginseng* (QBA29395.1), *Capsicum chinense* (PHU24077.1), *Nicotiana plumbaginifolia* (AAA34078.1), *Arabidopsis thaliana* (AT4G16260), and *Oryza sativa* (AAL40191.1), were used to create a phylogenetic tree. (**C**) Sub-cellular localization of *PgGH17* when transiently expressed in tobacco. Scale bars = 30 µm.

A *PgGH17* clone was obtained from the ginseng genome database, and its corresponding genomic DNA sequences were compared. *PgGH17* was located in two scaffolds, 2375 and 6649, containing one small exon and a long exon interrupted with one intron having an ORF size of 1128 bps (Figure 5A). The same sequence of Pg_S6649.4 and Pg_S2375.8 CDS was annotated in the ginseng genome, indicating its duplication. Although the scaffold 6649 sequence was not fully identified, the upstream 1000 bp promoter region of scaffold 2375 contains seven abscisic acid (ABA)-responsive cis-elements: DPBFCOREDCDC3 [18], ABRELATERD1 [19], ABREOSRAB21 [20], RYREPEATBNNAPA, and RYREPEATVFLEB4 [21]. Its homologs were searched for by NCBI BLASTx using the amino acid sequence, in order to understand the evolutionary relationship of the *PgGH17* gene. The amino acid sequence of PgGH17 shared 99.29% identity with the *P. notoginseng* b-1,3-glucanase protein (*PnGlu1*), 80.6% identity with the *Arabidopsis thaliana* beta-1,3-endoglucanase protein, 63.04% identity with the *Manihot esculenta* glucan endo-1,3-beta-glucosidase, basic vacuolar isoform protein, 61.93% identity with the *Hevea brasiliensis* glucan endo-1,3-beta-glucosidase, basic vacuolar isoform-like protein, 63.81% identity with the *Eucalyptus grandis* lichenase protein, and 82.93% identity with the *Oryza sativa* endo-1,3-*β*-glucanase protein (Figure 5B).

A GFP-fused PgGH17 protein was constructed using the 35S promoter and infiltrated tobacco epidermal cells, in order to identify the subcellular localization of PgGH17. Compared with the control, which showed GFP signals in the nucleus, cytosol, and plasma membrane, the signal of the PgGH17–GFP protein was only observed in the cytosol, cell periphery, and cell wall (Figure 5C). Similarly, the homologous gene in *P. notoginseng* (*PnGlu1*) was reported to be localized in the cell wall when transiently expressed in onion cells [22], but this requires further confirmation in *P. ginseng*. 

### 2.6. Salt Response in PgGH17-Overexpressing Arabidopsis Plants

Given the evidence that *PgGH17* transcript and protein levels are enhanced in ginseng by salinity stress, transgenic *A. thaliana* overexpressing *PgGH17* was generated to determine if its expression affects the salt tolerance of transgenic lines. Expression analysis was used to identify and select transgenic lines #13 and #14, possessing high expression of *PgGH17* transcripts and proteins (Figure S2). The growth phenotype of *PgGH17*-overexpressed *Arabidopsis* (*PgGH17ox*) was similar to the wild-type (WT) (Figure S2A). After obtaining the homozygous T2 generation, their growth in the NaCl-containing medium was tested (Figure S3). Looking at the *Arabidopsis* sensitivity to salt stress, NaCl concentrations ranging from 50–150 mM were used for the germination test and phenotypic measurements. Seed germination was not significantly different between the NaCl-free medium and the 50 mM NaCl medium (Figure S3A–C), but the germination ratios of *PgGH17ox* #13 and #14 were higher than WT at 100 and 150 mM NaCl concentrations (Figure S3D,E). 

**Figure 6:**
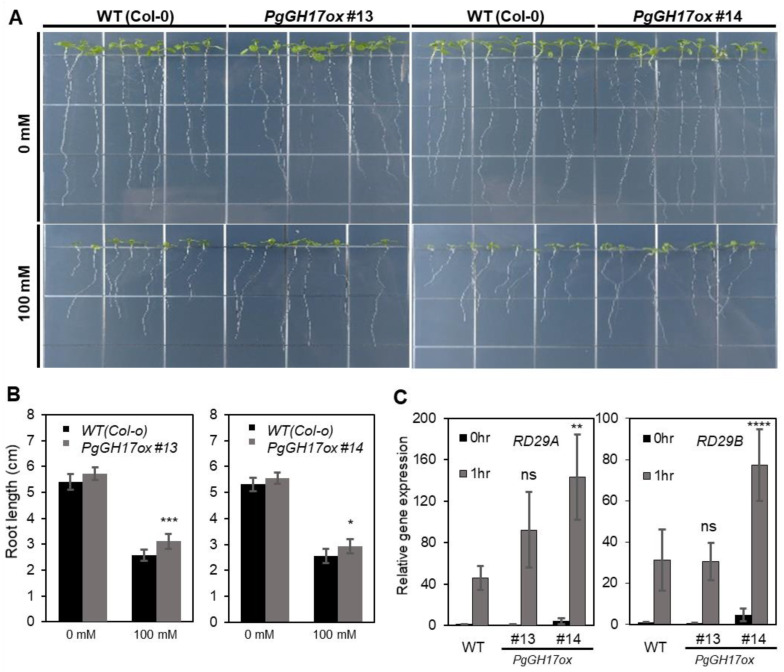
Salt tolerance of *PgGH17*-overexpressing plants. (**A**) Ten day-old seedlings with WT (Col-0) *PgGH17ox* were grown in ½ MS media with 100 mM NaCl. (**B**) Primary roots were photographed and analyzed with ImageJ software (https://imagej.nih.gov/ij, USA, accessed on 16 March 2022). The raw data were statistically analyzed using the Student’s *t*-test, and *p*-values were obtained using at least 35 seedlings per treatment. The error bars represent SE. (**C**) Induction of RD29A and RD29B salt-responsive genes after 1 h 150 mM NaCl treatment in HA-GHs overexpression lines. Asterisks indicate a significant difference between WT and *PgGH17*-overexpressing plants. (ns indicates no significance, * *p* < 0.05; ** *p* < 0.01; *** *p* < 0.001; **** *p* < 0.0001; the Student’s *t*-test).

The root growth inhibition was lower in *PgGH17ox* #13 and #14 under 100 mM NaCl stress treatment, while the root growths of WT and *PgGH17ox* were similar under normal conditions (Figure 6A). A statistically quantified result that compared the root length of transgenic lines to the WT in response to salt stress (Figure 6B) showed that the root lengths of *PgGH17ox* #13 and #14 were significantly longer than WT at 100 mM NaCl. 

The transcript levels of genes involved in the salt stress signaling pathways were examined to investigate the molecular mechanism of PgGH17 underlying the salt tolerance in transgenic *A. thaliana*. The homologous *RD29A* and *RD29B* genes are abiotic stress marker genes that are upregulated by various abiotic stresses, especially salinity and drought. [23,24]. The expression of the salt-detoxifying *salt overly sensitive* (*SOS*) was not altered. In contrast, the expression of *RD29A* and *RD29B* was induced by salt stress, which was even more enhanced in overexpressing lines than in the WT (Figure 6C), confirming that the heterologous expression of *PgGH17* results in the development of a salt-tolerant phenotype. 

## 3. Discussion

Understanding the molecular mechanism underlying the salt stress response is essential to limit the salt stress-induced damage in plants. Thus far, only a limited number of salinity stress-related genes have been identified and characterized in ginseng. This study identified the salinity stress-responsive proteins using a high-throughput proteomics approach, which revealed PgGH17. The functional roles of the identified PgGH17 were confirmed in a model plant, *A. thaliana,* using a transgenic approach. Although further experimentations are required to confirm the functional role of PgGH17 in salt stress tolerance in *P. ginseng*, overexpressed PgGH17 contributes to salt stress tolerance in *Arabidopsis.*

### 3.1. Activation of ROS Scavenging by Oxidative Stress

The accumulation of ROS during salinity stress causes damage to the plant cells [25,26]. In this study, a time-dependent accumulation of H_2_O_2_ and a membrane peroxidation product, MDA, was observed in ginseng leaves in response to salt stress (Figure 1B,C). Moreover, the enhanced activities of antioxidant enzymes were also observed in ginseng leaves (Figure 1D–F), suggesting the activation of antioxidant machinery to detoxify the stress-induced ROS. In addition, the proteomics results showed an increased abundance of superoxide dismutase (SOD) in response to salt stress in ginseng leaves (Table S1). SOD converts superoxide radicals to H_2_O_2_ [27], and CAT and APX decompose H_2_O_2_ to water [28]. On the other hand, this study showed that CAT and APX exhibit different activity trends during salt stress (Figure 1D,E). CAT and APX perform the same action, but APX scavenges ROS, mostly in the apoplastic space [29,30,31]. These results suggest that ROS-detoxification in ginseng occurs in various organelles.

### 3.2. Protein Metabolism in Response to Salt Stress

Salt stress generally inhibits protein synthesis [32]. Correspondingly, a decreased abundance of ribosomal proteins was also observed in ginseng leaves (Table S1). In addition, diverse heat shock proteins (Hsp), such as Hsp70, Hsp 70-interacting protein 1, and Hsp81-1, as well as other molecular chaperons, such as the TCP-1/cpn60 chaperonin family, peptidyl–prolyl cis-trans isomerase, and ATPases, were also identified in our analysis (Table S1). Hsps and chaperones are stress-response proteins involved in protein folding, assembly, translocation, and degradation. Previous studies showed that Hsp70 is expressed in response to various stresses and assists in the refolding of denatured or aggregated proteins, and Hsp70 overexpression could enhance tolerance to salt stress [33]. 

Plants utilize proteasome complexes to induce the degradation of unwanted proteins during abiotic stress, and the amino acids generated from the degradation of these proteins are utilized for the de novo synthesis of stress-related proteins [34]. The 26S proteasome is a protein complex involved in apoptosis and development [35] and is interconverted with the 20S proteasome to regulate *Arabidopsis’* tolerance to salt stress [36]. This study identified a proteasome subunit beta 1, a structural protein constituting the proteasome 20S [37] in C_5, showing an increased abundance in response to salt stress in ginseng leaves (Table S1). In addition, the expression of proteasome subunit beta 1 was increased even at the transcript level under salt conditions (Figure 4A–C). This result suggests that ginseng, as *A. thaliana*, resists salt stress through the interconversion of proteasomes 26S and 20S. 

### 3.3. Physiological Response of Ginseng to Salinity Exposure

Stress conditions damage the photosynthetic machinery and cause a decrease in photosynthetic efficiency [38]. The abundance of the ribulose-1,5-bisphosphate carboxylase-oxygenase (RuBisCO) large subunit decreased in response to salt stress in ginseng leaves. RuBisCO is a crucial enzyme of the Calvin cycle and catalyzes the first major step of carbon fixation. Previous studies reported a decreased abundance of the RuBisCO small subunit in response to salt stress [39]. In contrast to RuBisCO, an increased abundance of other photosynthesis-related proteins was observed in ginseng leaves during salt stress, such as oxygen-evolving enhancer (OEE) 1, photosystem II manganese-stabilizing protein (PsbP) 2, chlorophyll A-B binding protein (CAB), and RuBisCO activase (RCA) (Table S1). OEE1 and 2 are components of the PSⅡ oxygen-evolving complex (OEC). The increased abundance of OEE has been observed in several plants under salt stress conditions, which may be necessary to maintain oxygen evolution [40]. Low stromal CO_2_ induces the inactivation of RuBisCO by binding inhibitory sugars [41]. RCA is a member of the AAA + family and has various chaperone-like functions. In addition, RCA is also involved in removing inhibitory sugars from the active site of RuBisCO. Therefore, these proteins are believed to increase salt tolerance by preventing photosynthesis inhibition. Based on these results, ginseng leaf photosynthesis tends to be inhibited by salt stress. On the other hand, a possible tolerance mechanism also takes place to overcome it in parallel by increasing the abundance of other photosynthesis-related proteins.

To adapt to salt stress, plants’ energy metabolism changes, and the energy from normal growth and metabolism is diverted into synthesizing compounds that participate in stress tolerance. This study identified multiple glycolysis-related proteins: phosphoglucomutase (PGM), fructose-bisphosphate aldolase class 1 (FBPA), phosphoglycerate kinase (PGK), pyruvate kinase family protein, and enolase, showing salinity-induced abundances (Table S1), ATP synthase subunit, alcohol dehydrogenase, and malate dehydrogenase exhibited increased or decreased abundance at different time-points of salt stress in ginseng leaves (Table S1). Glycolytic enzymes produce energy and intermediate sugars for the biosynthesis of other metabolites, particularly amino acids, to cope with salt stress. Previous studies showed that the abundance of PGM is altered in response to diverse stress conditions, such as nutrient deficiency, drought, and fungal infection [42]. A PGM-like protein also exhibited salt stress-induced abundance in tomatoes [43]. An increased abundance of PGM was also observed in salt-treated ginseng leaves (Table S1). In addition to glycolysis, changes in fatty acid metabolism have also been reported during salt stress in different plants [44]. In particular, previous reports have indicated that the changes in fatty acyl chains affect plant salt tolerance [45,46]. A decreased abundance of acetyl Co-enzyme A carboxylase carboxyltransferase alpha subunit was identified (Table S1), suggesting that the desaturation process of fatty acids in ginseng is also changed by salt stress.

Ion transport helps determine salt stress tolerance in plants [47]. Different ion transporters are required to limit the entry of ions into the cells and to export excess ions outside the cytosol. Studies on the abiotic stress tolerance of plants through the overexpression of ion transporters have been steadily progressing [48,49]. This study identified a voltage-gated K^+^ channel *β* subunit and a voltage-dependent anion channel 1 that changed significantly in response to salt stress in ginseng leaves. A previous study reported that the voltage-gated K^+^ channel β subunit overexpression induced salt tolerance in sweet potatoes [50]. Voltage-dependent anion channel 1 is involved in salt stress tolerance in *A. thaliana* [51]. This shows that the ion transport mechanism of ginseng also changes to resist salt stress. Therefore, the proteins involved in energy metabolism and ion exchange are expected to be associated with salt tolerance, and to play an essential role in energy production and osmotic balance.

### 3.4. Role of PgGH17 in Salt Stress Tolerance in Ginseng

Emerging evidence suggests that maintaining the cell wall integrity is critical for the adaptation to high salinity [52]. In response to stresses, plants need to adjust the chemical and mechanical properties of their cell walls and need to recognize the damaged cell wall. In addition, the cytoskeleton is also remodeled rapidly to maintain cell turgor when subjected to salt stress. Here, the abundance of the essential cytoskeletal components, such as cellulose synthase-like protein D4, decreased in response to salt stress in ginseng leaves (Table S1), indicating that cell remodeling is activated in ginseng to adapt to salt stress. 

PgGH17 and PgGH18 are *β*-1,3-glucanase and chitinase, respectively. Both glycosyl hydrolase proteins play essential roles in cell wall remodeling [53]. Here, an increased abundance of these cell wall remodeling proteins under salt stress was noted, further indicating cell wall remodeling in ginseng as a response to salt stress. A recent study reported that PgGH18 plays a role in plant salt tolerance by inducing cell wall modifications, required for osmotic tolerance [54]. Chitinase and *β*-1,3-glucanase are involved in the plant defense response by hydrolyzing the cell walls of fungal and insect pathogens [55,56]. Recently, a PgGH17 homolog (*PnGlu1*), identified in *P. notoginseng*, provided strong resistance to *Fusarium* in transgenic tobacco plants [57]. *PnGlu1* was also induced by various stresses and hormone signals, including H_2_O_2_ [57]. Similar observations were also reported in rice, where OsGNL1 exhibited enhanced accumulation, both at the transcript and protein levels, in the root and shoot in response to drought and ABA [58]. In addition, NaCl, ABA, and H_2_O_2_ were reported to be up-regulated by a sugarcane *β*-1,3-glucanase D family gene (ScGluD2) [56]. In *Arabidopsis*, *β*-1,4-endoglucanase, named KORRIGAN1, was demonstrated to be an integral part of the cellulose synthase complex of the primary cell wall and was required for root elongation under salt stress [59,60]. 

Overall, these results suggest a crucial role of PgGH17 in the salt stress tolerance (Figure 6 and Figure S3) of ginseng. We did not detect significant ROS alteration in PgGH17ox compared with WT, indicating that the salt tolerance is not dependent on ROS mechanisms. The mechanisms could possibly induce cell wall modifications [61]. Further studies to identify the cell wall components modified by PgGH17 or signaling networks will be required to shed light on the exact role of PgGH17-mediated salt stress tolerance in plants.

## 4. Materials and Methods

### 4.1. Plant Materials and qRT-PCR

Ginseng (*P. ginseng* Meyer) leaves, cultivar ‘Yeonpung’, were obtained from the National Institute of Horticultural and Herbal Science, Rural Development Administration (RDA) at Eumseong-gun, South Korea (36° N, 127° E). Two year-old ginseng plants were grown in a greenhouse at an average temperature of 22.5 ± 2.5 °C and a humidity of 50 ± 10%. The cultivated ginseng was transferred to a hydroponic culture and acclimatized for two days before the salt stress treatment. Subsequently, 20 ginseng plants per treatment were exposed to a 250 mM NaCl solution, and samples were harvested at 0, 24, 72, and 96 h after treatment. After harvesting the leaves and petioles (leaf-attached stalk), each tissue was homogenized to a fine powder with liquid nitrogen and stored at −80 °C for analysis.

The total RNA was extracted from ginseng leaf samples with the RNeasy plant mini kit (Qiagen, Hilden, Germany), according to the manufacturer’s instructions. Briefly, reverse transcription reactions were carried out using PrimeScript reverse transcriptase and a genomic DNA eraser (Takara, Kusatsu, Japan). Quantitative real-time PCR (qRT-PCR) was performed in three replicates on a CFX96 cycler (BioRad, CA, USA) using an iTaq Universal SYBR Green Supermix (BioRad, CA, USA), according to the manufacturer’s instructions. Table S3 lists the primers used. The relative fold differences in the templates’ abundances were determined for each sample. The threshold cycle (Ct) value for each gene-specific gene was normalized to the Ct value for *β*-actin and calculated relative to a calibrator using the algorithm 2^−Δ^CT.

### 4.2. Lipid Peroxidation and Hydrogen Peroxide Assays

A lipid peroxidation assay was carried out by measuring the concentration of MDA according to a previous report [62]. Briefly, 0.2 g of the leaf sample was homogenized in 2 mL 10% trichloroacetic acid (TCA) and centrifuged at 12,000 rpm for 10 min at 4 °C to collect the clear supernatant. Sequentially, an equal volume of 0.6% thiobarbituric acid (TBA) in supernatant and mixture was heated in a 95 °C water bath for 30 min, and cooled quickly in an ice bath. After centrifugation at 12,000 rpm for 10 min at 4 °C, the absorbance of the mixture was determined at 532 and 600 nm. The nonspecific absorbance at 600 nm was subtracted from that at 532 nm, and the concentration of MDA was calculated using this adjusted absorbance and the extinction coefficient of MDA (155 mM^−1^cm^−1^).

H_2_O_2_ was quantified using the Amplex^®^ Red Hydrogen Peroxide/Peroxidase Assay Kit (A2218, Thermo Fisher Scientific, MA, USA) according to the manufacturer’s instruction and a previous report [63], where 10-acetyl-3,7-dihydroxyphenoxnazine with horseradish peroxidase (HRP) was used to measure H_2_O_2_. Briefly, 0.1 g of ground sample was homogenized in 1× reaction buffer (50 mM sodium phosphate pH 7.4), and was centrifuged at 12,000 rpm for 5 min at 4 °C. The clear homogenate was mixed with an equal volume of working reagent, containing 100 μM 10-acetyl-3,7-dihyfroxyphenoxnazine and 0.2 U/mL of HRP. The mixture was then incubated for 30 min at room temperature. The fluorescence was measured using a microplate reader with excitation and emission wavelengths of 571 nm and 585 nm, respectively. The H_2_O_2_ concentration was calculated in salt stress-treated ginseng leaves using the standard curve generated by the H_2_O_2_ concentration.

### 4.3. Determination of Antioxidant Enzyme Activities

APX, CAT, and DHAR activities were analyzed using a modification of a previously reported method [64]. The crushed frozen fresh ginseng leaf sample (0.2 g) was homogenized in 2 mL of 50 mM potassium phosphate buffer (pH 7.0) and subjected to centrifugation at 12,000 rpm for 10 min at 4 °C for collection of the top phase. The APX activity was measured by 290 nm absorption by mixing 1.9 mL of reaction buffer (25 mM potassium phosphate, pH 7.0; 0.25 mM L-ascorbic acid (AsA); 0.1 mM ethylene-diamine-tetraacetic acid (EDTA); 0.1 mM H_2_O_2_) with 0.1 mL of top phase. The DHAR activity was measured by 265 nm absorption by mixing 1.9 mL of DHAR reaction buffer (50 mM potassium phosphate, pH 7.8; 0.1 mM dehydroascorbic acid (DHA); 2.5 mM glutathione (GSH); 0.1 mM EDTA) with 0.1 mL of the top phase. For the CAT activity, 1.9 mL of CAT reaction buffer (50 mM potassium phosphate, pH 7.0; 10 mM H_2_O_2_) was measured at 240 nm absorbance. 

### 4.4. Total Protein Extraction Using Ginseng Leaves

The total leaf proteins were isolated, as previously reported [65]. Briefly, 1 g of fine ground ginseng leaf powder was homogenized with 5 mL of ice-cold Tris-Mg/NP-40 extraction buffer (0.5 M Tris–HCl, pH 8.3; 2% (*v*/*v*) Nonidet P-40 (NP-40); 20 mM MgCl_2_; 0.07% (*v*/*v*) *β*–mercaptoethanol) and centrifuged at 12,000 rpm for 10 min at 4 °C. The collected supernatant was mixed with the same volume of tris-saturated phenol (pH 7.6) and subjected to centrifugation at 12,000 rpm for 10 min at 4 °C, for the collection of lower-phase-containing proteins. The lower phase was incubated with four volumes of 0.1 M ammonium acetate dissolved in MeOH for 1 h at −20 °C to collect protein pellets. The resulting pellets were washed with 0.1 M ammonium acetate dissolved in MeOH and 80% (*v*/*v*) acetone containing 0.07% (*v*/*v*) *β*–mercaptoethanol, and then stored at −20 °C for further analysis.

### 4.5. In-Solution Trypsin Digestion, Peptide Desalting, and BPRP Fractionation

Protein digestion was carried out using a filter-aided sample preparation (FASP) approach, as described previously [66,67]. Briefly, acetone-precipitated protein (400 μg) was dissolved in 40 μL of denaturation buffer (4% sodium dodecyl sulfate (SDS) and 100 mM dithiothreitol (DTT) in 100 mM triethylammonium bicarbonate (TEAB); pH 8.5), followed by sonication for 3 min and heating at 99 °C for 30 min. The denatured protein was diluted 10-fold with urea buffer (8 M urea in 100 mM TEAB; pH 8.5) and transferred to a 30K spin filter (Merck Millipore, Darmstadt, Germany). SDS was removed by buffer exchange with urea buffer and cysteine alkylation, with 200 μL of alkylation buffer (50 mM iodoacetamide (IAA); 8 M urea in 100 mM TEAB; pH 8.5). After alkylation, the trypsin solution, dissolved in 50 mM TEAB containing 5% acetonitrile (ACN) (enzyme to substrate ratio (*w*/*w*) of 1:50), was added and incubated at 37 °C overnight. The concentration of obtained peptides was measured using the Pierce Quantitative Fluorometric Peptide Assay kit (Thermo Scientific, Waltham, MA, USA), according to the manufacturer’s instructions [65]. The digested peptides were subsequently applied to desalting HLB OASIS columns and basic pH reversed-phase (BPRP) fractionation using an in-house developed stage tip, respectively, as reported elsewhere [68].

### 4.6. LC-MS/MS Analysis

Label-free quantitative proteomic analysis of ginseng leaves was performed as described elsewhere [69,70]. Each peptide fraction was re-dissolved in solvent-A (2% ACN and 0.1% formic acid), followed by reversed-phase chromatography separation using a UHPLC Dionex UltiMate^TM^ 3000 instrument (Thermo Fisher Scientific, Waltham, MA, USA). To separate the peptides, a trap column (Thermo Scientific, Acclaim PepMap 100 trap column, 100 μm × 2 cm, nanoViper C18, 5 μm, 100 Å) and analytic column (Thermo Scientific, Acclaim PepMap 100 capillary column, 75 μm × 15 cm, nanoViper C18, 3 μm, 100 Å) were used. The peptides were separated with 150 min of a nonlinear gradient of solvent-B from 2% to 35% (100% ACN and 0.1% formic acid), followed by 40 to 95% for 10 min, 95% solvent-B for 5 min, and 2% solvent-B for 15 min. The electrospray ionization source was coupled with a quadrupole-based mass spectrometer, QExactive™ Orbitrap High-Resolution Mass Spectrometer (Thermo Fisher Scientific, MA, USA). The resulting peptides were electrosprayed through a coated silica-emitted tip (Scientific Instrument Service, NJ, Amwell Township, USA) at an ion spray voltage of 2000 eV. A data-dependent mode for the 15 most abundant peaks (Top15 method) was used for the measurement of the mass spectra of each sample. The precursor ions were acquired with a resolution of 70,000 at 200 m/z in a mass range of 350–1800 m/z. The automatic gain control (AGC) target value was 3 × 10^6^, and the isolation window for MS/MS was 1.2 m/z. Ion activation/dissociation with higher energy C-trap dissociation (HCD) scans was acquired at a resolution of 35,000 and normalized collision energy (NCE) value of 32. The AGC target value for MS/MS was 2 × 10^5^. The maximum ion injection time for the survey scan and MS/MS scan was 30 ms and 120 ms, respectively. The proteomics data were deposited to the ProteomeXchange Consortium via the PRIDE partner repository, with the dataset identifier PXD038524 [71].

### 4.7. LFQ Data Analysis

The obtained MS spectra data were analyzed using MaxQuant software (ver. 2.0.3.0), as described before [72]. Each replicate of the MS/MS spectra of ginseng samples was cross-referenced against the *Panax ginseng* database (http://ginsengdb.snu.ac.kr, 59,352 entries, accessed on 19 January 2020) using MaxQuant software, integrated with the Andromeda search engine [73]. For label-free quantification (LFQ) data analysis, default precursor mass tolerances were set as 20 ppm for the first search, and 4.5 ppm for the following ones, by Andromeda in MaxQuant. In addition, 0.5 Da of a product mass tolerance with a maximum of two missed cleavages of tryptic digestions was used for the LFQ data search. Carbamidomethylation of cysteine residues was selected for fixed modification. The acetylation and oxidation of lysine and methionine residues, respectively, were chosen as variable modifications. The false discovery rate (FDR), set at 1% for peptide identifications, was determined based on a reverse nonsense version of the original database.

Perseus software (ver. 1.6.15.0) was used for further statistical analysis of the LFQ data [74]. The missing value imputation of the LFQ intensities of the identified proteins from a normal distribution (width: 0.3, downshift: 1.8), hierarchical clustering analysis (HCA), and multiple sample test (Benjamini-Hochberg FDR threshold of 0.05), as well as the identification of significant differences in protein abundance (>1.5-fold change) among the different sample sets, were carried out using Perseus software [68,74]. GO enrichment and functional classification were performed using AgriGO v2.0 [75] and MapMan software [76], respectively.

### 4.8. Construction of the Plant Expression Vector and Transformation

*35S:HA-GH17* transgenic lines were generated by amplifying the *HA-GH17* sequence using T-blunt *GH17* and T-blunt GH18 vector templates. The hemagglutinin (HA) tag sequence and linker (GGGGS) sequence were added in front of the coding sequences using *GH17-F* and *GH17-R* primers described in Table S3. The *HA-GH17* PCR product was cloned into the pENTR D-TOPO vector (Invitrogen, Carlsbad, CA, USA) for gateway cloning, and after verifying the sequence, HA-GHs inserts were transferred to the pH7WG2 binary vector using lambda integrase/exisionase (ELPIS, Daejeon, Korea) to generate the *35S:HA-GH17* destination vector. Constructed plasmids harboring GH17 overexpression cassettes were introduced to the *Agrobacterium tumefaciens* strain GV3101 using the electroporation method. The floral dip method was performed to introduce the *35S:HA-GH17* vector into *Arabidopsis thaliana* from *Agrobacterium*. The transgenic plants were screened based on the expression levels of the GH17 gene.

For subcellular localization analysis, PgGH17 CDS was cloned into a pGreen vector fused with C-terminal green fluorescence protein (GFP). The constructs were transformed into *A. tumefaciens* strain GV3101 and used for *Nicotiana benthamiana* infiltration. Three days after infiltration, GFP fluorescence was observed by confocal laser scanning microscopy (K1-Fluo, Nanoscope systems, Daejeon, Korea) using 488/505–530 nm excitation/emission filter sets.

### 4.9. Arabidopsis Material and Growth Condition

This study used the *A. thaliana* Columbia-0 (Col-0) ecotype as the control. *A. thaliana* seeds were surface-sterilized with chlorine gas, generated from a solution combining 100 mL of 4% sodium hypochlorite and 6 mL HCl for six hours in a desiccator. After sterilization, seeds were sown on agar plates containing 1/2 Murashige and Skoog (MS) medium (Duchefa Biochemie, Haarlem, The Netherlands) supplemented with 1% (*w*/*v*) sucrose, and 0.8% (*w*/*v*) plant agar (Duchefa Biochemie, Haarlem, The Netherlands); the pH of the medium was adjusted to 5.8. All *A. thaliana* seedlings were grown under long-day conditions (16 h light/8 h dark) at 22 °C with 100–120 µmol m^−2^s^−1^ fluence rates for gene and protein expression analysis. In the case of primary root length analysis, 35 seeds of T2 or T3 homozygous lines were germinated in different concentrations (mock, 50, 100, and 150 mM) of NaCl-supplemented MS medium, according to reports [77,78,79].

### 4.10. Arabidopsis Expression Examination

The total RNA was isolated from 10 day-old seedlings using a HigeneTM Total RNA Prep kit (Biofact, Daejeon, Korea). In the case of the salt-responsive gene assay, 150 mM NaCl solution was sprayed onto 10 day-old WT and *PgGH17*ox seedlings and sampled after 1 h. The extracted RNA samples were quantified from the measured A260 nm/A280 nm ratios for protein contamination and from the A260 nm/A230 nm ratios for reagent contamination, using BioPhotometer Plus (Eppendorf, Hamburg, Germany). Subsequently, 2 µg of the total RNA was used for first-strand cDNA reverse transcription using LyofactTM RT Pre-mix (Biofact, Daejeon, Korea). The relative transcripts were analyzed by a CFX96 qPCR machine (Bio-rad Laboratories, Hercules, CA, USA). The *IPP2* (*AT3G02780*) gene was used as an internal control to normalize relative gene expression levels. Table S3 lists the gene-specific primer (qGH17-F, qGH17-R, qIPP2-F, qIPP2-R, qRD29A-F, qRD29A-R, qRD29B-F, qRD29B-R) sequences.

Ten day-old seedlings grown on 1/2 MS media under long-day conditions were examined to detect the HA-GH17 protein. The whole protein of samples was extracted using extraction buffer (50 mM sodium phosphate buffer, pH 7.4; 100 mM NaCl; 10% (*v*/*v*) glycerol; 5 mM EDTA; 0.5% SDS; 1% NP-40; 0.5% sodium deoxycholate; 50 µM MG-132; complete protease inhibitor cocktail tablets (Roche, Basel, Switzerland)). The proteins were resolved in 12% SDS-PAGE gels and transferred to a nitrocellulose membrane (GE Healthcare, Milan, Italy). The HA-GH17 proteins were detected using an anti-HA (Roche, Basel, Switzerland) antibody, and actin proteins were detected using anti-actin (Wuhan, China, ABclonal) antibodies. In the case of actin, anti-mouse IgG peroxidase antibodies (Sigma, St Louis, MO, USA) were used to activate the immunoreaction. The ECL select^TM^ Western blotting detection reagent (Amersham, Buckinghamshire, UK) was used to visualize immunoreactive signals using a cooled CCD camera (ChemiDoc touch, Bio-rad Laboratories, Hercules, CA, USA).

### 4.11. Salt Tolerance Test of PgGH17-Expressed Arabidopsis

Stratified WT and *PgGH17*ox seeds were sown in 1/2 MS medium with 1.2% (*w*/*v*) bactoagar, containing 100 mM NaCl, and then grown vertically using a square plate in long-day conditions for 10 days. The primary roots of 10 day-old seedlings were photographed and analyzed using ImageJ software (https://imagej.nih.gov/ij, accessed on 16 March 2022, USA). At least eight roots of WT and transgenic plants were measured for each replicate, the raw data were analyzed statistically using a Student’s *t*-test, and the *p*-value was obtained. Eight biological replicates were used to assess reliability. 

### 4.12. Statistical analysis

The mean ± standard deviation values of each replicate were examined to validate the statistical significance of each dataset. The error bars indicate the standard error. For multiple samples, one-way ANOVA tests were evaluated using Dunnett’s multiple comparisons test, and a Student’s *t*-test was applied to compare the significant differences between two samples using GraphPad Prism software (San Diego, CA, USA). The term ns indicates no significance, * indicates the significance of *p* < 0.05, ** indicates the significance of *p* < 0.01, *** indicates the significance of *p* < 0.001, and **** indicates the significance of *p* < 0.0001.

## 5. Conclusions

A label-free quantitative proteomic analysis was used to identify the salt-responsive proteins in ginseng leaves. This study identified many salt stress-responsive proteins related to photosynthesis, protein degradation, cell wall remodeling, sugar biosynthesis, sugar degradation, and sugar accumulation. The *PgGH* family proteins involved in cell wall metabolism and defense response had an increased abundance during salt stress treatment. In particular, the qRT-PCR analysis revealed the accumulation of the *PgGH17* gene (>10-fold) in a similar manner, as shown at the proteome level. Furthermore, *PgGH17*ox lines showed cell wall repair/recovery and the activation of ROS metabolism under salt stress conditions. Overall, these results provide new evidence supporting the critical role of the PgGH17 protein in salt stress tolerance in ginseng. The results reported here may be helpful for further understanding the molecular mechanism of the salt stress response in ginseng leaves/plants.

## Data Availability

The proteomics data were deposited to the ProteomeXchange Consortium via the PRIDE partner repository, with the dataset identifier PXD038524.

## Supplementary Materials

The supporting information can be downloaded at: https://www.mdpi.com/article/10.3390/ijms24043693/s1.
