# Supplementary material for: Proteomic Analysis Reveals a Critical Role of the Glycosyl Hydrolase 17 Protein in Panax ginseng Leaves under Salt Stress
Source: Int J Mol Sci. 2023 Feb 12;24(4):3693. doi: 10.3390/ijms24043693 (PMC9965409; doi:10.3390/ijms24043693)
Supplement: Supplementary file 1 [file ijms-24-03693-s001.zip › Supplementary Figures.pptx]

## Slide 1
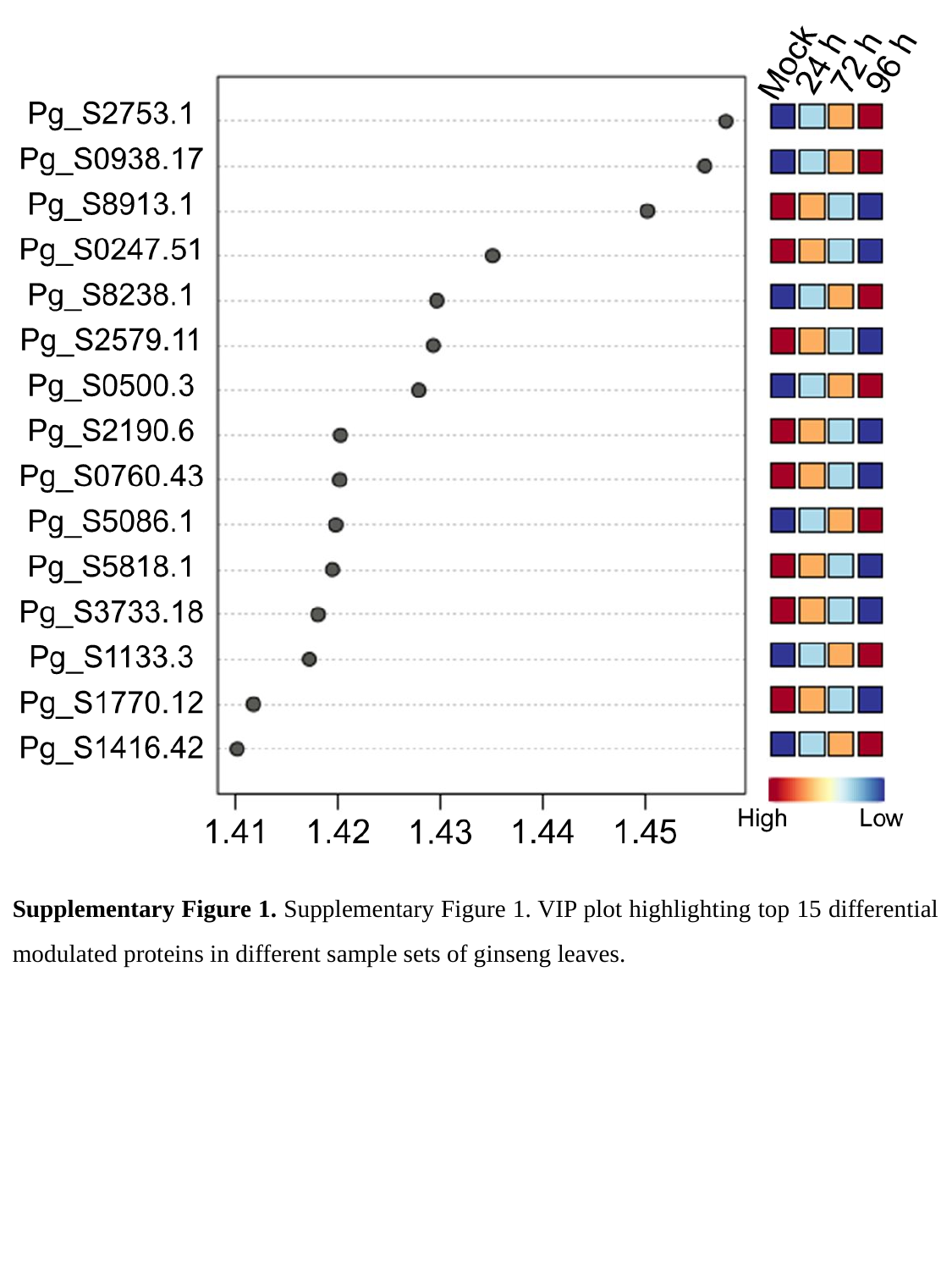

Supplementary Figure 1. Supplementary Figure 1. VIP plot highlighting top 15 differential modulated proteins in different sample sets of ginseng leaves.

## Slide 2
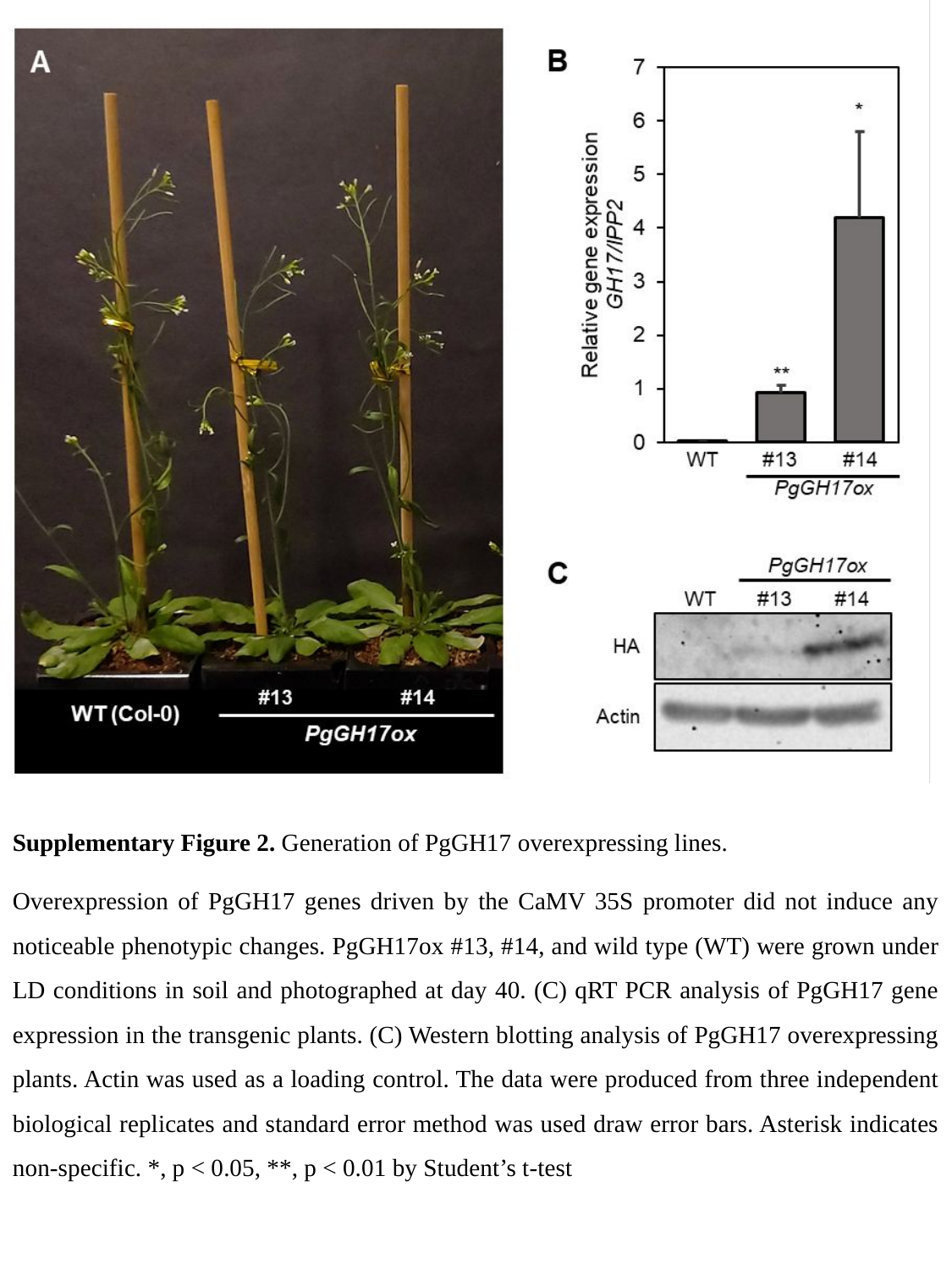

Supplementary Figure 2. Generation of PgGH17 overexpressing lines.
Overexpression of PgGH17 genes driven by the CaMV 35S promoter did not induce any noticeable phenotypic changes. PgGH17ox #13, #14, and wild type (WT) were grown under LD conditions in soil and photographed at day 40. (C) qRT PCR analysis of PgGH17 gene expression in the transgenic plants. (C) Western blotting analysis of PgGH17 overexpressing plants. Actin was used as a loading control. The data were produced from three independent biological replicates and standard error method was used draw error bars. Asterisk indicates non-specific. *, p < 0.05, **, p < 0.01 by Student’s t-test

## Slide 3
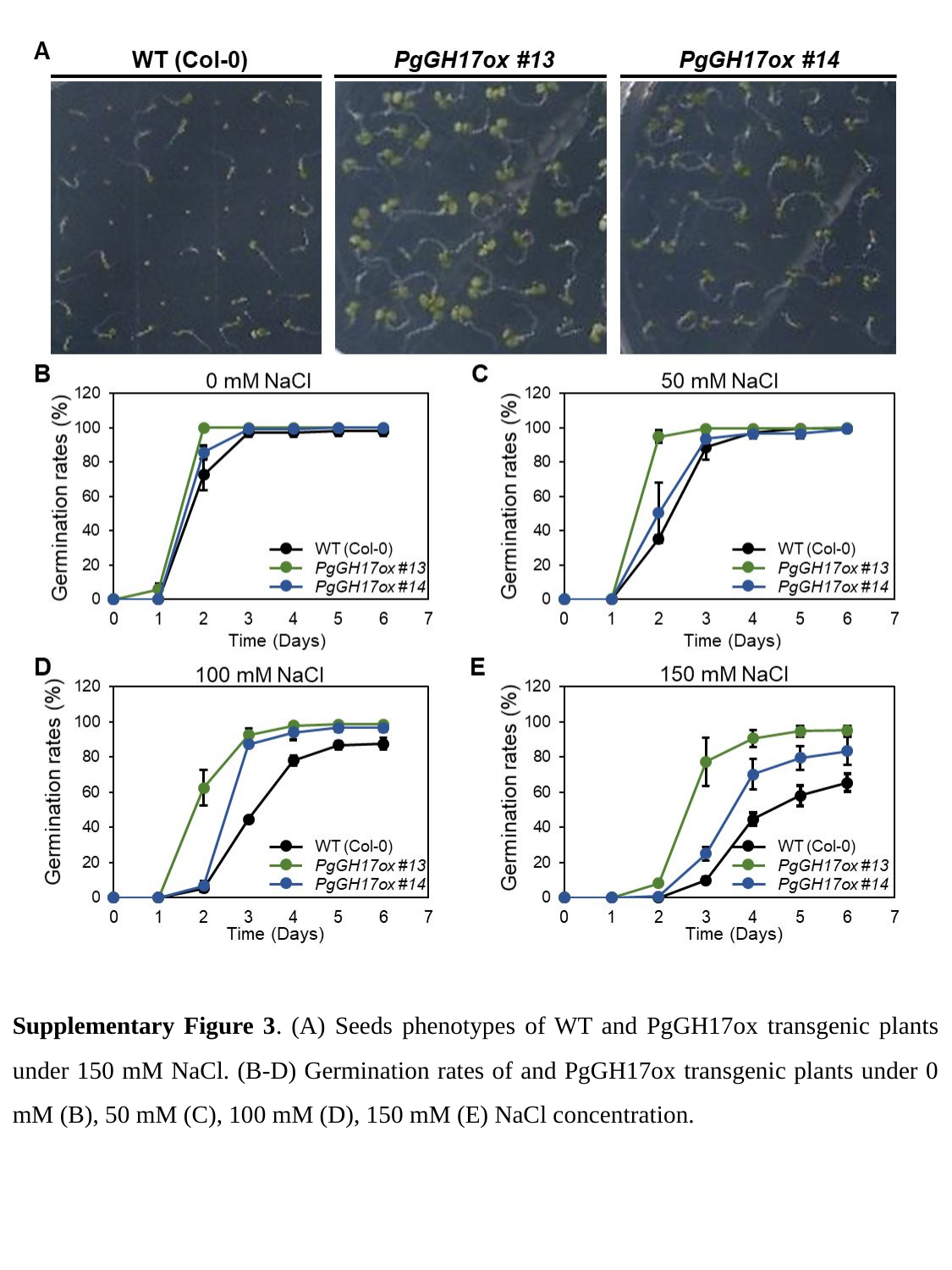

Supplementary Figure 3. (A) Seeds phenotypes of WT and PgGH17ox transgenic plants under 150 mM NaCl. (B-D) Germination rates of and PgGH17ox transgenic plants under 0 mM (B), 50 mM (C), 100 mM (D), 150 mM (E) NaCl concentration.
